# Acupuncture combined with balloon dilation for post-stroke cricopharyngeal achalasia: A meta-analysis of randomized controlled trials

**DOI:** 10.3389/fnins.2022.1092443

**Published:** 2023-01-12

**Authors:** Jing Luo, Bingjing Huang, Huiyan Zheng, Zeyu Yang, Mingzhu Xu, Zhenhua Xu, Wenjun Ma, Run Lin, Zitong Feng, Meng Wu, Shaoyang Cui

**Affiliations:** ^1^Department of Rehabilitation, Shenzhen Hospital of Guangzhou University of Chinese Medicine, Shenzhen, Guangdong, China; ^2^Guangzhou University of Chinese Medicine, Guangzhou, Guangdong, China; ^3^Clinical Medical College of Acupuncture Moxibustion and Rehabilitation, Guangzhou University of Chinese Medicine, Guangzhou, Guangdong, China; ^4^Department of Rehabilitation, Shenzhen Hospital, Southern Medical University, Shenzhen, Guangdong, China; ^5^Guangdong Provincial Hospital of Chinese Medicine, Guangzhou, Guangdong, China

**Keywords:** acupuncture, balloon dilation, stroke, cricopharyngeal achalasia, meta-analysis

## Abstract

**Background:**

The purpose of this study was to systematically evaluate the effectiveness of acupuncture combined with balloon dilatation in patients with post-stroke cricopharyngeal achalasia (CPA) according to the effective rate, videofluoroscopy swallowing study (VFSS) score and standardized swallowing function assessment scale (SSA) score through Meta-analysis.

**Methods:**

English and Chinese language literature published before July 24,2022 were searched in ten electronic databases. The identified articles were screened, data were extracted, and the methodological quality of the included trials was assessed. Using RevMan 5.4.1 software to perform Meta-analysis.

**Results:**

10 studies with 517 patients with post-stroke CPA were included. Meta-analysis showed that the effective rate of the experience group was higher than that of the control group [OR = 0.62; 95% CI (2.32, 13.05); *I*^2^ = 0%; *p* = 0.0001]. Compared to the control group, the SSA score was lower in the experience group [MD = −4.22; 95% CI (−4.57, −3.87); *I*^2^ = 42%; *p* < 0.00001]. In terms of VFSS scores, the experience group showed greater efficacy differences than control group [MD = 1.53; 95% CI (1.32, 1.75); *I*^2^ = 0%; *p* < 0.00001]. The subgroup analysis of VFSS score based on the average course of disease (<1 month vs. ≥1 month) showed no significant difference. The subgroup analysis based on average age (>60 years vs. ≤60 years) showed the VFSS score of the experience group was significantly higher than that of the control group, and the effect may be better in the subgroup older than 60 years. The subgroup analysis based on the treatment course (>30 days vs. ≤30 days) showed the VFSS score of the experience group was significantly higher than that of the control group, and the effect may be better in the subgroup the treatment course>30 days.

**Conclusion:**

Acupuncture combined with balloon dilatation may be an effective method for treating post-stroke CPA. Compared with balloon dilatation, acupuncture combined with balloon can significantly improve the swallowing function of patients, and it is also effective for patients of different courses, ages, and treatment course, while patients over 60 years old and the treatment course over 30 days may have better clinical outcomes.

## Introduction

Post-stroke dysphagia (PSD) is a common complication after stroke (Takizawa et al., 2016), and on the verge 5.7% of them are attributable to cricopharyngeal achalasia (CPA) (Regan et al., 2014; Yang et al., 2018). CPA usually cause severe dysphagia, with serious complications which could lead to an increase in medical expenses and mortality, a decrease in quality of life, etc (Kocdor et al., 2016). Currently, balloon dilation is a regular treatment of post-stroke CPA (Dou et al., 2012; Dewan et al., 2020). Through repeated mechanical traction and dilation, balloon dilation can relax the cricopharyngeal muscle (CPM) and improve swallowing (Suntrup et al., 2015). However, simple balloon dilatation involves a long period, which is not always effective at solving the problems of delayed swallowing, weak swallowing, poor swallowing endurance and aspiration. Simple balloon dilatation is difficult to achieve satisfactory results, especially for severe ones.

Being a relatively simple, inexpensive, and safe treatment (Yang et al., 2016), acupuncture has been recommended by the World Health Organization (WHO) as an alternative and complementary method for treating stroke (Belskaya et al., 2020). A systematic review of 6,010 patients also showed that acupuncture could improve post-stroke dysphagia (Ye et al., 2017). In addition, combination of acupuncture and rehabilitation provides a novel strategy for the clinical rehabilitation of stroke (Tang et al., 2022). Le Peng et al. found that compared to rehabilitation therapy alone, the combined therapy is more effective (Peng et al., 2018). In recent years, clinical studies have reported better results of acupuncture combined with balloon dilation than simple balloon dilation in treating post-stroke CPA. However, no reliable objective evidence exists to support its exact efficacy. Therefore, we conducted this systematic analysis of randomized controlled trial (RCT) studies to assess the clinical efficacy of acupuncture combined with balloon dilation on post-stroke CPA and try to provide evidence for the clinical treatment of post-stroke CPA.

## Materials and methods

### Search strategies

**Figure 1:**
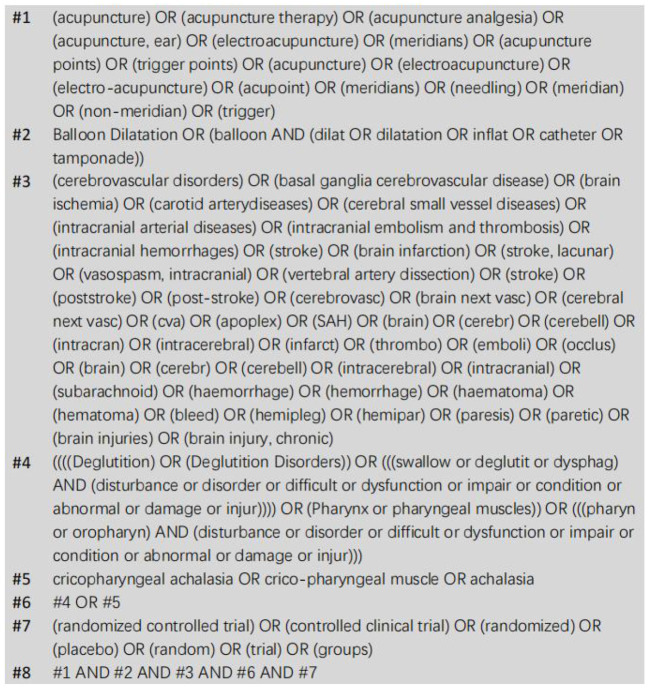
PubMed retrieval strategy.

Between the establishment and July 24, 2022, PubMed, Embase, Web of Science, The Journal of Alternative and Complementary Medicine, Medline, The Cochrane Library, CBM, CNKI, VIP and WanFang Data were systematically searched in both Chinese and English. We searched acupuncture combined with balloon dilatation in the treatment of post-stroke CPA with the search term “swallow disturbance” or “cricopharyngeal achalasia” and “Balloon Dilatation” and “acupuncture” and “stroke.” A “randomized controlled trial” search was conducted. As an example, the search strategy for PubMed is shown in Figure 1. References and review articles with potential relevance studies were manually examined. A PROSPERO registration number (CRD42022350411) was assigned to this study.

### Inclusion and exclusion criteria

To select the studies for inclusion in this meta-analysis, the following criteria were used: (1) RCTs; (2) Post-stroke patients with CPA (confirmed by videofluoroscopy swallowing study); (3) Patients enrolled in studies with age <80 years; (4) Acupuncture combined with balloon dilatation was used in the experimental group, while simple balloon dilatation was used in the control group, and the researchers provided original data or sufficient information about dysphagia that occurred pre- and posttreatment in experimental trials and control trials. Exclusion criteria: (1) Identical publications; (2) The original research data was not provided and cannot be obtained by contacting the original author; (3) A control group did not receive simple balloon dilation (acupuncture and other measures were also included); (4) Inclusion criteria were not met by some publications.

### Outcome measures

According to this study, the outcome measures were: effective rate, standardized swallowing function assessment scale (SSA), and Videofluoroscopy Swallowing Study (VFSS).

### Data extraction and quality assessment

Data were independently extracted by two well-trained evaluators to review the original text. The disagreements were solved by the third author's assistance. A study's publication year and first author were included in its characteristics. Among the patient characteristics were age, sample size, intervention measures, treatment course and average course of disease. The efficiency rate, SSA score, and VFSS score were also calculated. The study's methodological quality was assessed according to the risk of bias tool described in the Cochrane system evaluator's handbook 5.1.0 (Jpt, 2011). The risk of bias was assessed by random sequence generation, allocation concealment, blinding of personnel and participants, blinding of outcome assessors, selective reporting, incomplete outcome data, and other potential risks.

### Statistical analysis

For all statistical analyses, Rev Man 5.4.1 was used. For dichotomous variables, risk ratios (RR) or odds ratio (OR) and 95% confidence interval (CI) were used as statistical tools for efficacy analysis and effect sizes, respectively. There were mean difference (MD) and 95% CI for continuous variables. *I*^2^ statistic was used as a measure of heterogeneity indicating the percentage of total variability in a set of effect sizes caused by true heterogeneity (Huedo-Medina et al., 2006). For high, moderate, and low heterogeneity, *I*^2^ values of 75, 50, and 25% were used (Ampt et al., 2018). A fixed-effects model for data pooling was used if the *I*^2^ statistic was below 50%, which meant that the included studies displayed acceptable heterogeneity. Whenever the *I*^2^ statistic was above 50%, the random-effects model was employed, followed by subgroup analysis or sensitivity analysis (Qiao et al., 2022).

## Results

### Characteristics of studies

**Figure 2:**
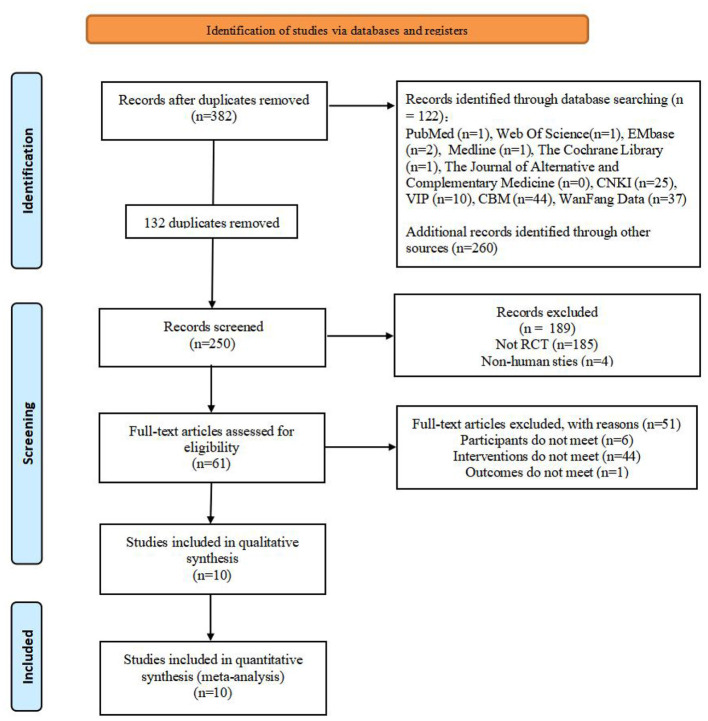
PRISMA flowchart of the study selection process. RCT, randomized controlled trials; acupuncture combined with balloon dilation: Cricopharyngcal achalasia.

In the initial retrieval, 382 articles totally were found. After layer-by-layer screened, 10 articles were finally included in the meta-analysis (He, 2017; Yang and Lei, 2017; Zhang, 2017, 2019; Cao et al., 2019; Li et al., 2019; Fan et al., 2020; Gao, 2020; Luo et al., 2020; Long et al., 2021). Study selection, literature screening and reasons for exclusion are shown in the PRISMA diagram (Figure 2). According to Table 1, the primary characteristics of the included studies are listed.

**Table 1:** Characteristics of the randomized controlled studies.

**Study**	**N (T/C)**	**Average age ±SD (T/C, YrS)**	**Average course of disease ±SD (T/C)**	**Interventions**		**Treatment course**	**Outcomes**
				**T**	**C**		
Cao et al. (2019)	30/30	61 ± 5/61 ± 5	30.86 ± 0.52/30.57 ± 0.64 (d)	Acupuncture + Balloon Dilation	Balloon Dilation	4 wks	②
Fan et al. (2020)	33/33	67.31 ± 5.36/66.58 ± 5.83	1.35 ± 0.59/1.29 ± 0.42 (mo)	Acupuncture + Balloon Dilation	Balloon Dilation	③	
Gao (2020)	24/24	52.68 ± 4.75/50.85 ± 4.69	4.82 ± 0.24/4.45 ± 0.16 (wks)	Acupuncture + Balloon Dilation	Balloon Dilation	4 wks	①-③
He (2017)	31/31	59.65 ± 8.58/59.00 ± 8.72	133.65 ± 29.52/131.55 ± 28.34 (d)	Acupuncture + Balloon Dilation	Balloon Dilation	1 mo	① ③
Li et al. (2019)	15/15	56 ± 8/57 ± 8	2.5 ± 1.6/2.5 ± 1.4 (mo)	Acupuncture + Balloon Dilation	Balloon Dilation	6 wks	③
Long et al. (2021)	30/30	60.53 ± 10.61/59.93 ± 12.89	21.73 ± 18.07/22.07 ± 16.74 (d)	Electroacupuncture + Balloon Dilation	Balloon Dilation	4 wks	① ②
Luo et al. (2020)	34/33	63.3 ± 7.3/62.6 ± 8.1	28.2 ± 6.8/29.4 ± 5.8 (d)	Acupuncture + Balloon Dilation	Balloon Dilation	4wks	① ③
Yang and Lei (2017)	24/24	60 ± 8/60 ± 5	4.57 ± 0.64/4.86 ± 0.52 (wks)	Tongue Acupuncture + Balloon Dilation	Balloon Dilation	4 wks	②
Zhang (2017)	20/20	63.56 ± 8.03/65.06 ± 11.09	2.72 ± 2.44/3.01 ± 2.88 (mo)	Acupuncture + Balloon Dilation	Balloon Dilation	-	③
Zhang (2019)	18/18	61 ± 9/59.3 ± 10.1	23.9 ± 16.1/25.3 ± 18.7 (d)	Acupuncture + Balloon Dilation	Balloon Dilation	20 d	① ③

① clinical treatment effects; ②SSA; ③VFSS.

T, experimental group; C, control group; min, minutes; d, days; wk, weeks; m, months; y, years.

### The effective rate

**Figure 3:**
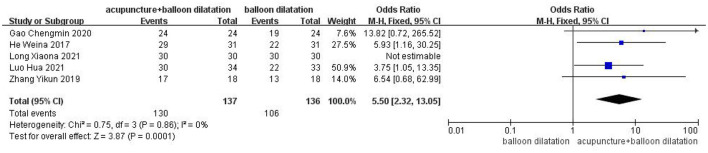
Forest plot for the effective rate.

Five studies were included in total. The findings of the fixed effect model analysis revealed that the efficacy rate of acupuncture combined with balloon dilatation was statistically significantly greater than that of simple balloon dilatation [OR = 5.50, 95%CI (2.32, 13.05), *p* = 0.0001] (Figure 3).

### Standardized swallowing function assessment scale (SSA)

**Figure 4:**
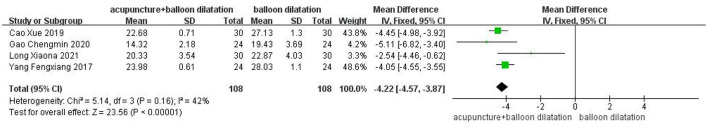
Forest plot for the SSA score.

Four studies were included in total. The fixed effect model analysis revealed that in the combination of acupuncture with balloon dilatation group, the SSA score was statistically significantly lower than in the simple balloon dilatation group [MD = −4.22, 95%CI (−4.57, −3.87), *p* < 0.00001] (Figure 4).

### Videofluoroscopy swallowing study (VFSS)

**Figure 5:**
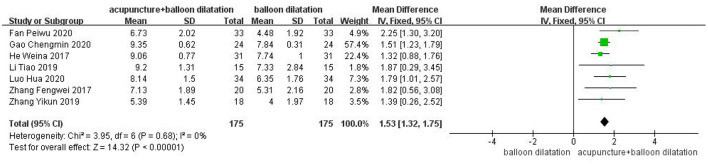
Forest plot for the VFSS score.

7 studies were included in total. The findings of the fixed effect model analysis revealed that the VFSS score of the acupuncture combined with balloon dilatation group was statistically significantly higher than that of the simple balloon dilatation group [MD = 1.53, 95%CI (1.32, 1.75), *p* < 0.00001] (Figure 5).

### Subgroup analyses

Based on VFSS scores, we performed analysis of 2 subgroups, including the average course of disease (<1 month vs. ≥1 month) and the average age (>60 years vs. ≤60 years).

### The average course of disease

**Figure 6:**
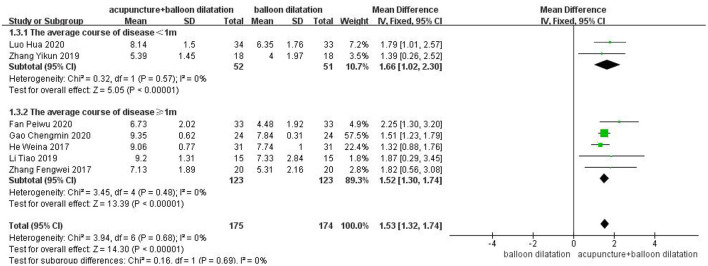
Forest plot for subgroup analysis for the average course of disease: the average course of disease <1 m vs. the average course of disease ≥1 m.

A total of two studies were included in the subgroup with an average course of disease was <1 month, and analyzed by fixed model showed that the VFSS score of acupuncture combined with balloon dilatation group was higher than that of simple balloon dilatation group [MD = 1.66, 95%CI (1.02, 2.30), *p* < 0.00001]. However, a total of 5 studies were included in subgroups with an average course of disease was ≥1 month, and analyzed by fixed effect model showed that the VFSS score in acupuncture combined with balloon dilatation group was also higher than that of simple balloon dilatation group [MD = 1.52, 95%CI (1.30, 1.74), *p* < 0.00001]. And acupuncture combined with balloon dilation showed a greater significant effect in patients whose course of disease is <1 month, with low heterogeneity between groups (*I*^2^ = 0%, *p* = 0.69) (Figure 6).

### The average age

**Figure 7:**
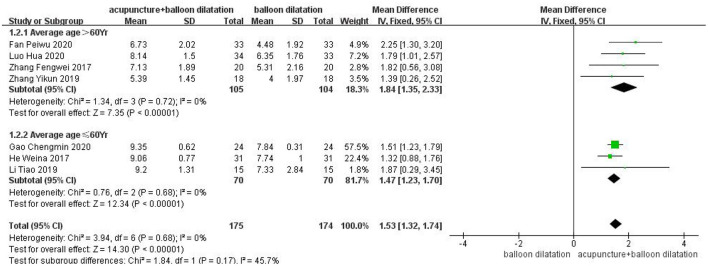
Forest plot for subgroup analysis for the average age: the average age >60 Yr vs. the average age ≤60 Yr.

A total of four studies were included for subgroup with an average age over 60, and analyzed by fixed effect model. Average age subgroup analysis demonstrated that the VFSS score in acupuncture combined with balloon dilatation group was higher than that in the simple balloon dilatation group [MD = 1.84,95%CI (1.35, 2.33), *p* < 0.00001]. And three studies for subgroup with an average age ≤ 60 years showed the same result [MD = 1.47, 95%CI (1.23, 1.70), *p* < 0.00001]. However, the result in the >60 years group revealed a higher effect size than the control conditions. Among groups, there is moderate heterogeneity (*I*^2^ = 45.7%, *p* = 0.17) (Figure 7).

### The treatment course

**Figure 8:**
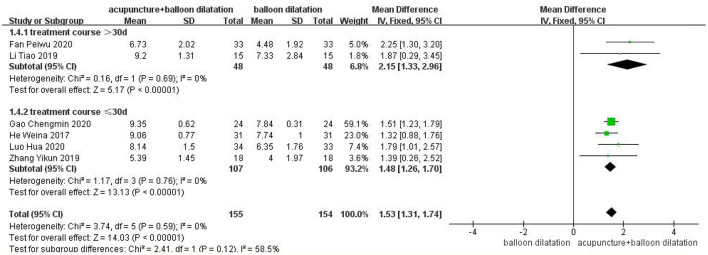
Forest plot for subgroup analysis for the treatment course: the treatment course >30 d vs. the treatment course ≤30 d.

A total of two studies were included for the subgroup with the treatment course over 30 days, and analyzed by fixed effect model. Treatment course analysis demonstrated that the VFSS score in acupuncture combined with balloon dilatation group was higher than that in the simple balloon dilatation group [MD = 2.15, 95%CI (1.33, 2.96), *p* < 0.00001]. And 4 studies for subgroup with the treatment course ≤30 days showed the same result [MD = 1.48, 95%CI (1.26, 1.70), *p* < 0.00001]. However, the >30 days group result revealed a higher effect size than the control conditions. Among groups, there is moderate heterogeneity (*I*^2^ = 58.5%, *p* = 0.12) (Figure 8).

### Risk of bias for independent studies

**Figure 9:**
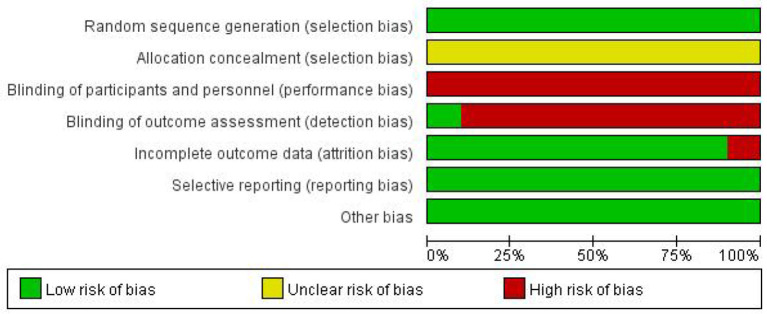
Performance of each type of bias in all studies.

**Figure 10:**
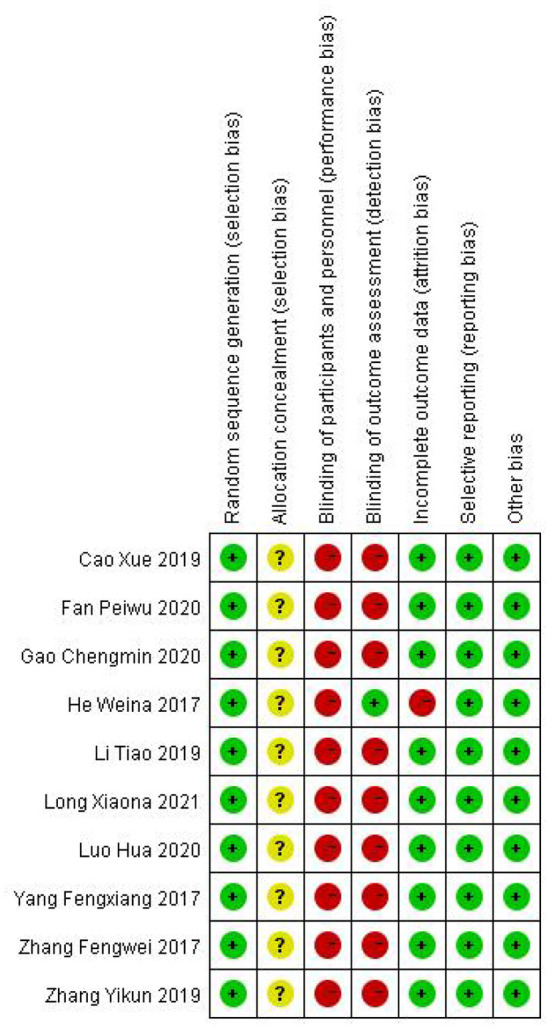
Summary plot of bias in all studies.

Bias risk assessment results are shown in Figures 9, 10.

## Discussion

Post-stroke CPA is currently treated mainly with balloon dilatation, acupuncture neuromuscular electrical stimulation (NMES) through the skin, cricopharyngeal myotomy, taking botulinum toxin injections, etc. However, the clinical efficacy of the monotherapy is not satisfactory (Mason et al., 1998; Freed et al., 2001; Brigand et al., 2007; Bülow et al., 2008; Gallas et al., 2010; Kos et al., 2010; Rofes et al., 2013; Kocdor et al., 2016; Knigge and Thibeault, 2018; Lin, 2018). It is often argued that combined therapies might be more effective (Xie et al., 2021). Currently, balloon dilatation has become a commonly used method for treating post-stroke CPA (Dou et al., 2012). Patients suffering from post-stroke CPA can also benefit from acupuncture in terms of swallowing function, and acupuncture has fewer adverse reactions than other treatments (Jia et al., 2009; Dou et al., 2013; Arnold et al., 2016; Han and Gao, 2017; Rajahthurai et al., 2022). But there is no high-quality evidence for the effect of acupuncture combined with balloon dilation. Therefore, we conducted this Meta-analysis with different subgroups to explore the efficacy of acupuncture combined with balloon dilatation and simple balloon dilatation on post-stroke CPA, trying to provide more effective treatment for clinic. The results of this meta-analysis showed that acupuncture combined with balloon dilatation is a superior treatment comparing to simple balloon dilatation in treating post-stroke CPA. It is also effective for patients at different courses of the disease (less than one month or longer than one month), different ages (over 60 years old and under 60 years old) and different treatment course (over 30 days and under 30 days), while patients over 60 years old, and the treatment course over 30 days may have the better clinical outcome.

Acupuncture combined with balloon dilation may play their respective advantages. Post-stroke CPA manifests as tonic contraction or incoordination of the CPM (Luan et al., 2021). CPM is a significant component of the upper esophageal sphincter (UES) and it is innervated by the recurrent laryngeal nerve and the pharyngeal plexus vagus nerve (Lierse, 1992). Balloon dilatation provides sensory input to the swallowing center while expanding the CPM, strengthens the damaged cortical and subcortical connection, promotes nerve remodeling, as well as improving swallowing abilities (Dou et al., 2012). It is reported that, balloon dilatation has a certain effect in the early stage of the disease. However, the effect in the late follow-up gradually declines as time goes on, only 36% of the patients' symptoms have been relieved (Müller et al., 2018). Moreover, research had shown that, Simple balloon dilatation has poor effect on aspiration and dysphagia of some refractory post-stroke CPA patients with long course of the disease. It is difficult to solve the problems such as delayed start of swallowing, weakness of swallowing muscles, poor swallowing tolerance and aspiration. Many patients still need to rely on nasogastric tube to eat (Li et al., 2019). Guo and Malik (2019) found that acupuncture could stimulate glossopharyngeal, sublingual, and vagus nerves, regulate the excitability of the swallowing-related cerebral center, enhance brain plasticity and promote swallowing function. In addition, acupuncture can stimulate swallowing related muscles and regulate the tension of the CPM (Huang et al., 2020; Chen et al., 2022). Yao et al. (2022). found that acupuncture could improve the movement and sensory function of the pharynx, improve swallowing activation, and reducing the incidence of infiltration-misaspiration. This may be why acupuncture combined with balloon dilatation is superior to simple balloon dilatation for treating post-stroke CPA.

In the assessment of dysphagia, the VFSS is regarded as the gold standard. Based on VFSS assessment, subgroup analysis for different courses of the disease (<1 month or longer than 1 month) both revealed that there was a statistically significant difference between acupuncture combined with balloon dilatation group and simple balloon dilatation group. In comparison with balloon dilatation alone, acupuncture combined with balloon dilatation group showed a larger effect in improving the swallowing function of patients. It indicates that acupuncture combined with balloon dilatation may be a more effective treatment for different courses of post-stroke CPA. After the stroke, the patient's brain structure and connectivity have changed. Related studies have found that, the plasticity of the brain provides a basis for rehabilitation after stroke (Xie et al., 2022), as time goes on, the brain tissue has a certain ability to repair itself (Hermann and Chopp, 2012). Acupuncture combined with balloon dilatation may promote this compensation and repair mechanism, and reorganize the brain function of stroke patients (Zhang et al., 2021), thus improving swallowing function, promoting rapid recovery of patients, and shortening the disease's course. At present, research on the various stages of post-stroke CPA is still insufficient. Due to data limitations, we were unable to complete the detailed analysis of acupuncture combined with balloon dilatation in the acute phase, recovery phase, and sequelae phase.

According to the subgroup analysis of average ages, regardless of whether they were older or younger than 60, there was no difference between the two groups, the VFSS score of the acupuncture combined with balloon dilatation group was significantly higher than the balloon dilatation group (*I*^2^= 45.7%, *p* = 0.17). Wilmskoetter et al. (2019). found that age is a negative predictor of the recovery for PSD. Interestingly, our meta-analysis found that acupuncture combined with balloon dilation had a larger effect size in the subgroup of older adults (MD = 1.84 > MD = 1.47). Acupuncture could promote the recovery of neuromuscular function, which may overcome the adverse effects of age on the repair of nerve injury, so that the damaged function could be better repaired. Shi et al. (1995) found the same effect in acupuncture treating for other diseases.

The subgroup analysis of the treatment course indicated a possible cumulative benefit of the combined therapy, with the prolongation of treatment duration, this suggested the dose-effect relationship of acupuncture combined with balloon dilation, and consistent with the previous study (Peng et al., 2018; Li et al., 2022; Xu et al., 2022). However, we cannot judge the best treatment course because of the limited literature. An implication of our result is that, in treating CPA, the treatment course of acupuncture combined with balloon dilation is preferably greater than 30 days.

Safety is a significant indicator in acupuncture studies, and we should have included it in our research. However, in extracting data from the original article, we found that only one piece mentioned safety indicators (adverse events), and its statistical adverse events were 0. In other articles, we did not mention to extract relevant data on security. Therefore, we cannot conduct further data analysis. This also reminds us to pay attention to the observation of safety indicators when doing research about acupuncture in the future.

Nonetheless, we should concede that this study has a few restrictions. First, in addition to age, disease duration, and treatment course, the patient's lesion location, stroke severity, and etiology can significantly impact on the prognosis of these patients. We should have included them in our research. However, in the original articles, the baseline data collection for this disease was primarily biased toward various functional evaluation tables. Most of the above specific vital factors were not mentioned or differentiated. We tried to contact the original author, but they have not replied. This meta-analysis may be affected by differences in the etiology, location of lesions, and stroke severity among the studies. Second, among the included articles, only one was in English, suggesting a bias in language selection. Finally, although the included articles had a good homogeneity, it should be noted that the evidence strength was still low due to the high risk of bias in the included studies, and the example size was small. Therefore, the conclusions should be interpreted with more caution, and more high-quality large-sample RCT literature analyses are urgently needed to confirm the conclusions.

## Conclusion

Post-stroke CPA may be treated effectively with acupuncture combined with balloon dilatation. In comparison with balloon dilatation alone, acupuncture combined with balloon dilatation can significantly improve the gulping capability of patients, and it is also effective for patients at different courses of the disease (less than one month or longer than one month), different ages (over 60 years old and under 60 years old) and different treatment course (over 30 days and under 30 days), while patients over 60 years old and the treatment course over 30 days may have the better clinical outcome.

## Data availability statement

The original contributions presented in the study are included in the article/supplementary material, further inquiries can be directed to the corresponding author.

## Author contributions

JL responsible for article retrieval and writing. BH and HZ responsible for literature screening, data extraction, and statistical analysis. JL, BH, and HZ are joint first authors and contributed equally. ZY assists BH and HZ in their work. RL, ZF, WM, and MW responsible for bias risk assessment. MX and ZX responsible for the guidance on modification of this paper. SC responsible for the review of articles and ensuring that all listed authors have approved the manuscript before submission. All authors contributed to the article and approved the submitted version.
